# Gender differences and determinants of prevalence, awareness, treatment and control of hypertension among adults in China and Sweden

**DOI:** 10.1186/s12889-020-09862-4

**Published:** 2020-11-23

**Authors:** Ailiana Santosa, Yue Zhang, Lars Weinehall, Genming Zhao, Na Wang, Qi Zhao, Weibing Wang, Nawi Ng

**Affiliations:** 1 Department of Epidemiology and Global Health, Faculty of Medicine, Umeå University, 90187 Umeå, Sweden; 2 Global Public Health, School of Public Health and Community Medicine, Institution of Medicine, Sahlgrenska Academy, University of Gothenburg, 41390 Gothenburg, Sweden; 3 School of Public Health, Key Laboratory of Public Health Safety of Ministry of Education, Fudan University, Shanghai, 200032 China

**Keywords:** High blood pressure, Hypertension diagnosis, Hypertension treatment, Inequalit

## Abstract

**Background:**

Failure to promote early detection and better management of hypertension will contribute to the increasing burden of cardiovascular diseases. This study aims to assess the gender differences in the prevalence, awareness, treatment and control of hypertension, together with its associated factors, in China and Sweden.

**Methods:**

We used data from two cross-sectional studies: the Västerbotten Intervention Program in northern Sweden (*n* = 25,511) and the Shanghai survey in eastern China (n = 25,356). We employed multivariable logistic regression to examine the socio-demographics, lifestyle behaviours, and biological factors associated with the prevalence, awareness, treatment and control of hypertension.

**Results:**

Men had a higher prevalence of hypertension (43% in Sweden, 39% in China) than their female counterparts (29 and 36%, respectively). In Sweden, men were less aware of, less treated for, and had less control over their hypertension than women. Chinese men were more aware of, had similar levels of treatment for, and had less control over their hypertension compared to women. Awareness and control of hypertension was lower in China compared to Sweden. Only 33 and 38% of hypertensive Chinese men and women who were treated reached the treatment goals, compared with a respective 48 and 59% in Sweden. Old age, impaired glucose tolerance or diabetes, a family history of hypertension or cardiovascular diseases, low physical activity and overweight or obesity were found to increase the odds of hypertension and its diagnosis.

**Conclusions:**

This study shows the age and gender differences in the prevalence, awareness, treatment and control of hypertension among adults in China and Sweden. Multisectoral intervention should be developed to address the increasing burden of sedentary lifestyle, overweight and obesity and diabetes, all of which are linked to the prevention and control of hypertension. Development and implementation of the gender- and context-specific intervention for the prevention and control of hypertension facilitates understanding with regard to the implementation barriers and facilitators.

**Supplementary Information:**

The online version contains supplementary material available at 10.1186/s12889-020-09862-4.

## Background

In 2015, 24.1% of men and 20.1% of women in the world were diagnosed with hypertension, one of the primary non-communicable disease (NCD) risk factors [1]. While hypertension and hypertension related NCDs and their complications are mostly preventable, poor case identification and management have contributed to the worldwide epidemic of hypertension. Antihypertensive drugs can effectively reduce cardiovascular diseases (CVDs) attributed to essential hypertension, while changes in lifestyle such as salt reduction [2] and weight management [3] can lower blood pressure and prevent hypertension [4, 5]. Nevertheless, the majority of hypertensive patients are unaware of their hypertension, receive poor treatment and have poor control of their blood pressure and therefore do not meet the recommended therapeutic targets [6]. This is particularly challenging in many low-income and middle-income countries where the prevalence of NCD and hypertension is on the rise [1, 7, 8].

Sweden and China are two countries that have experienced an epidemiological transition, with a shift from a predominance of infectious diseases to the NCDs [9], with hypertension seen as one of the leading risk factors contributing to NCD deaths. A longitudinal study in northern Sweden showed a significant downward shift in the prevalence of hypertension among the middle-aged population of Västerbotten County between 1990 and 2010 [10]. The awareness and control of hypertension in Västerbotten County also improved with an increase from 46.5 to 69% and from 30 to 65%, respectively. Despite the improvement, the gender gap in the awareness of hypertension remained, with a higher proportion of women than men who were aware of their hypertension (61.7% vs 51.6%, respectively) [11]. A national survey of 1.7 million Chinese adults conducted in 2009–2010 reported that about 37% of adults were hypertensive. Among the hypertensives, about 36% were aware of their hypertension, only 23% were receiving treatment, and of only 5.7% had their hypertension controlled [12]. Another national level study in China showed differences in the prevalence of hypertension among Chinese men and women (31% vs 28%, respectively) [13].

Though there is an abundance of studies on the prevalence, awareness, treatment and control of hypertension in China [14], similar studies are lacking in Sweden [11, 15, 16]. The current study fills this gap by providing a comparative analysis between China and Sweden, as well as identifying gender differences between the factors related to the prevalence, awareness, treatment and control of hypertension. Even though both Sweden and China’s health systems aim to provide humanitarian-egalitarian health care for all of their population, the organisation and finance of the health systems in the two countries are quite different [17]. The primary health care system in Sweden fits well with the goals for long-term management of hypertension in the population. A recent study in Sweden shows the effectiveness of education and feedback strategies for physicians in implementing the clinical guidelines for hypertension management at a primary care level in Sweden [18]. The primary health care system in China, however, has a considerably poorer quality of processes and outcomes [19]. Two recent reviews indicated significant gaps in the management of hypertension and diabetes at a primary care level in China [19, 20]. Comparing these two countries which are currently at different stages of epidemiological transition and different health system functioning, using hypertension as an example, can provide information for the implementation of CVD prevention programme in these two settings.

## Methods

### Study design and population

This study used two cross-sectional datasets from the Västerbotten Intervention Program (VIP) in Västerbotten County in the northern part of Sweden and the Shanghai Survey in eastern China.

The VIP is one of the very few long-term CVD prevention programmes in the world that is integrated into routine primary health care (PHC) and combines low-risk population and high-risk individual strategies [21]. Since 1990, the VIP invites all county residents turning 40, 50 and 60 years old to a health examination that screens for CVD risk factors, along with offering a health dialogue at their PHC with a trained nurse to discuss strategies for the adoption of a healthy lifestyle. The VIP refers individuals identified as being at high risk to physicians for further evaluation and treatment. VIP participants complete the self-administrated health questionnaire, consisting of socio-demographic information, self-reported health and quality of life, lifestyle behaviour such as tobacco use, alcohol consumption and physical activity, and history of chronic diseases and medications. Physical measurements and blood sampling are also taken by nurses. In this study, we used the VIP data collected during 2014–2017 (*n* = 25,511 individuals). The overall VIP response rate was about 70%. Using the unique person number in Sweden, Statistics Sweden linked the VIP Data with the population register data on socioeconomic indicators. The Department of Epidemiology and Global Health at Umeå University maintains this anonymous linked dataset, which is known as the Edwin database.

The Shanghai survey, part of a cohort study conducted in eastern China [22], selected a representative sample of residents aged 35–64 years old who had been living in the area under study for more than five years. Recruitment took place between June 2016 and December 2017 and a multistage stratified clustered sampling method was used. At the first stage, we selected four communities (Zhongshan, Xinqiao, Sheshan and Maogang Town) in Songjiang District to represent different geographical regions and economic levels. At the second stage, one third of the committees/villages were selected randomly from each community. At the third stage, we selected adults aged 35–64 years randomly from each of administrative units selected in the second stage. A total of 25,356 individuals (a response rate of 95.8%) completed the electronic questionnaire which collected data on sociodemographic indicators, health behaviours (smoking, alcohol consumption, and physical activity), and history of chronic diseases. Community-licensed physicians conducted weight, height, blood pressure measurements and blood sampling. We obtained the hypertension treatment data from the Songjiang Health System Big Data Platform.

### Outcome variables: prevalence, awareness, treatment and control of hypertension

In both settings, blood pressure was taken in a sitting position using a digital sphygmomanometer (Omron®) on the right mid-arm at heart level, after resting for five minutes. Two (in Sweden) or three (in China) blood pressure measurements were taken at five-minute intervals, and the mean value of all measurements was calculated.

An individual was considered to be hypertensive if their systolic blood pressure (SBP) was ≥140 mmHg or diastolic blood pressure (DBP) ≥90 mmHg or if they were currently taking antihypertensives [23]. Individuals with hypertension who had never been informed by a doctor or healthcare professional were considered unaware of their hypertensive status. The prevalence of treatment of hypertension was estimated based on the self-reported use of an antihypertensive drug in the last 14 days (in the VIP) or an antihypertensive drug prescription (in Shanghai) among those who were aware of their hypertensive status. We calculated the prevalence of the control of hypertension as being the proportion of individuals who had SBP < 140 mmHg and DBP < 90 mmHg among those who were being treated.

### Socio-demographic variables

We recorded age groups at 40, 50 and 60 years for VIP participants and 35–44 years, 45–54 years, 55–64 years for Shanghai participants. We dichotomised marital status as ‘currently married’ (married and cohabiting) and ‘currently unmarried’ (unmarried, widow, divorced). We categorised education into ‘basic education’ (those who had completed nine years of compulsory education), ‘medium education’ (those who had completed twelve years of education) and ‘high education’ (those who had graduated from university).

### Lifestyle behaviours

#### Smoking

In the VIP data, smoking status was defined based on the question “Do you currently smoke?”. Based on the responses to this question, we grouped the participants as never smokers, ex-smokers (those who used to smoked daily or occasionally), and current smokers (those who smoke daily or occasionally at the time of the study). In the Shanghai survey, smoking status was based on the participants’ responses to two questions: “Have you ever smoked at least one cigarette every day for more than six months?” and “What is your current smoking status?”. We grouped smoking status into three categories of never smokers, ex-smokers and current smokers.

#### Alcohol consumption

In the VIP data, assessment of alcohol consumption was based on the question “How often do you drink alcohol?”. We defined current alcohol drinkers as those who drank alcohol at least 2–3 times in a week. In the Shanghai survey, alcohol consumption was assessed based on the question “Have you ever drunk alcohol at least three times a week for more than six months?”. We defined respondents who answered yes to this question as being current alcohol drinkers.

#### Physical activity

In the VIP, physical activity was assessed using questions based around modes of travel to and from work, activities during recreational time, and physical exercise in the last three months. Based on responses to these three questions, respondents were categorised as ‘sedentary’, ‘moderately active’ or ‘physically active’ [24]. In the Shanghai survey, physical activity was measured using the International Physical Activity Questionnaire (IPAQ) Short Form, which asks about walking, moderate-intensity activity and vigorous-intensity activity. Levels of physical activity were categorised as ‘high level’, ‘moderate level’ or ‘low level’ following the IPAQ guideline [25].

#### Cholesterol

The Shanghai Survey determined serum total cholesterol using enzyme colorimetry (Roche COBAS C501 automatic biochemical analyser), and we defined hypercholesterolemia as total serum cholesterol ≥6·2 mmol/L. The VIP used a Reflotron bench-top analyser (Roche Diagnostics) and defined hypercholesterolemia using the same threshold (serum cholesterol ≥6·2 mmol/L) and/or the use of a lipid-lowering medication during the previous 14 days.

#### Body mass index

Height and weight were measured in light indoor clothing and used to calculate body mass index (BMI) (kg/m^2^). In the VIP data, we categorised the BMI, based on the World Health Organisation (WHO) definitions, into three categories of ‘underweight and normal weight’ (BMI < 24.9 kg/m^z^), ‘overweight’ (BMI 25–29.9 kg/m^2^) and ‘obese’ (BMI ≥30 kg/m^2^) [26]. In the Shanghai data, we grouped the BMI based on Chinese criteria from the Working Group on Obesity in China (WGOC): BMI < 24 kg/m^2^ as ‘underweight and normal weight’, BMI 24–27.9 kg/m^2^ as ‘overweight’ and BMI ≥28 kg/m^2^ as ‘obese’ [27].

#### Diabetes

In the VIP data, raised blood glucose was defined as measured capillary fasting plasma glucose ≥7 mmolL-1, or 2-h plasma glucose ≥12.2 mmolL-1, or self-reported diabetes diagnosed by a health professional [28]. In the Shanghai data, diabetes was defined as HbA1c concentration of 6.5% (48 mmol/L) or more [29].

#### Family history of disease

Questions assessed whether any family member of the participant had suffered from either diabetes, hypertension or CVDs.

##### Statistical analyses

We presented the descriptive characteristics of socio-demographics, lifestyle behaviours, and biological factors for each country. The country-specific prevalence, awareness, treatment, and control of hypertension were all adjusted to age and education levels. We employed multivariable logistic regression analysis to examine the factors associated with the prevalence, awareness, treatment and control of hypertension in each country. All analyses were performed separately for men and women due to the different hypertension burden as well as the distribution of its determinants in both sexes. We conducted all statistical analyses in Stata Version 15.1 (StataCorp, College Station, Texas, USA) for the Swedish data, and on SAS Version 9.4 (SAS Institute, Inc., Cary, NC, USA) for the Shanghai data.

## Results

A total of 25,511 individuals aged 40, 50 and 60 years old participated in the VIP, while a total of 25,356 individuals aged 35–64 years were recruited in the Shanghai Survey. We excluded 8.3 and 5.1% of participants respectively who had missing values in the variables included in the analysis from each of the studies. A total of 23,409 in the VIP and 24,055 individuals in the Shanghai data were included in the subsequent analyses.

The characteristics of the two study populations (VIP participants in Sweden and Shanghai Survey participants in China, referred to subsequently as the Swedish and Chinese participants for simplicity) were quite distinct (Table 1). The majority of the study participants in China were 55–64 years old, currently married and of low education level. In contrast, about 33% of men and 48% of women who participated in Sweden had high education levels. Chinese men were more likely to smoke, drink alcohol and have low physical activity than the Swedish men. Diabetes was more prevalent in China (14.0% in men and 11.4% in women in China vs 5.6% in men and 3.6% in women in Sweden). Conversely, the prevalence of obesity was almost two times higher among men and women in Sweden than among their Chinese counterparts. The prevalence of hypercholesterolaemia was higher in Sweden (22.9% in men and 21.5% in women) than in China (6.7% in men and 10% in women). Chinese women were less likely to smoke, or drink alcohol compared to their Swedish counterparts (0.3% ever smoked and 0.8% drank alcohol vs 42.2% and 14.6%, respectively). In both settings, women reported a higher level of physical activity compared to men.

**Table 1:** Characteristics of the study participants in Eastern China and in Northern Sweden

Variables	Eastern China (***N*** = 24,055)		Northern Sweden (***N*** = 23,409)	
	Men (n, %)	Women (n, %)	Men (n, %)	Women (n, %)
Age				
35–44 / 40 year	1141 (12.3)	1901 (12.8)	3720 (32.0)	3657 (31.0)
45–54 / 50 year	3304 (35.7)	5946 (40.2)	4036 (34.7)	4084 (34.6)
55–64 / 60 year	4805 (52.0)	6958 (47.0)	3861 (33.2)	4051 (34.4)
Marital Status				
Currently married	8971 (97.0)	14,042 (94.8)	9077 (78.1)	9458 (80.2)
Currently unmarried	279 (3.0)	763 (5.2)	2540 (21.9)	2334 (19.8)
Education level				
High (13 and above)	409 (4.4)	520 (3.5)	3798 (32.7)	5697 (48.3)
Medium (10–12)	1642 (17.8)	1683 (11.4)	6915 (59.5)	5366 (45.5)
Low (up to 9)	7199 (77.8)	12,602 (85.1)	904 (7.8)	729 (6.2)
Smoking				
Never smokers	3617 (39.1)	14,761 (99.7)	7365 (63.4)	6818 (57.8)
Ex-smokers	679 (7.3)	8 (0.1)	2931 (25.2)	3727 (31.6)
Current smokers	4954 (53.6)	36 (0.2)	1321 (11.4)	1247 (10.6)
Current alcohol drinkers				
No	6238 (67.4)	14,688 (99.2)	9528 (82.0)	10,067 (85.4)
Yes	3012 (32.6)	117 (0.8)	2089 (18.0)	1725 (14.6)
Physical activity				
Low level	6615 (71.5)	9683 (65.4)	2174 (18.7)	1188 (10.1)
Moderate level	1958 (21.2)	4087 (27.6)	5518 (47.5)	5989 (50.8)
High level	677 (7.3)	1035 (7.0)	3925 (33.8)	4615 (39.1)
Body mass index				
Underweight /Normal	3693 (39.9)	7724 (52.2)	3475 (29.9)	5425 (46.0)
Overweight	4165 (45.0)	5382 (36.4)	5429 (46.7)	3864 (32.8)
Obese	1392 (15.0)	1699 (11.5)	2713 (23.4)	2503 (21.2)
Hypercholesterolemia	617 (6.7)	1487 (10.0)	2655 (22.9)	2540 (21.5)
Treatment for cholesterol	NA	NA	950 (8.2)	579 (4.9)
Fasting blood glucose				
Normal	6757 (73.1)	10,930 (73.8)	9458 (81.4)	9877 (83.8)
Impaired glucose tolerance	1202 (13.0)	2188 (14.8)	1501 (12.9)	1492 (12.7)
Diabetes	1291 (14.0)	1687 (11.4)	NA	NA
Diabetes, treated	NA	NA	374 (3.2)	246 (2.1)
Diabetes, untreated	NA	NA	284 (2.4)	177 (1.5)
Family history of diabetes	1115 (12.1)	2056 (13.9)	2458 (21.2)	3024 (25.6)
Family history of hypertension	4280 (46.3)	7442 (50.3)	2119 (18.2)	2414 (20.5)

The prevalence of hypertension among men was higher in Sweden than in China (42.7% vs 38.6%, respectively), as shown in Table 2. The prevalence of hypertension among women was, by contrast, higher among Chinese women than among Swedish women (35.8% vs 28.6%, respectively). Swedish men and women had a higher prevalence of awareness and control of hypertension than their Chinese counterparts. In China, only 33.2% and 37.6% of the treated hypertension cases among men and women, respectively, were controlled and reached the therapeutic goal. The corresponding numbers were higher at 47.6% and 58.7% in Sweden.

**Table 2:** Prevalence, awareness, treatment and control of hypertension among men and women in Eastern China and in Northern Sweden

	Eastern China (***n***= 24,055)				Northern Sweden (***n*** = 23,409)			
**Men**	**35–44 years (*n*** **= 1141)**	**45–54 years (*n*** **= 3304)**	**55–64 years (*n*** **= 4805)**	**Total (*n*** **= 9250)**	**40 years (*n*** **= 3720)**	**50 years (*n*** **= 4036)**	**60 years (*n*** **= 3861)**	**Total (*n*** **= 11,617)**
	**% (95% CI)**	**% (95% CI)**	**% (95% CI)**	**% (95% CI)**	**% (95% CI)**	**% (95% CI)**	**% (95% CI)**	**% (95% CI)**
Prevalence of hypertension in the population	30.0 (27.3–32.9)	38.5 (36.8–40.1)	41.5 (40.2–42.9)	38.6 (37.6–39.6)	25.2 (23.8–26.7)	43.5 (42.0–45.1)	62.4 (60.9–64.0)	42.7 (41.7–43.7)
Hypertensive people who were aware about their hypertension	35.0 (29.9–40.5)	45.9 (43.2–48.6)	54.4 (52.2–56.6)	50.2 (48.5–51.8)	44.6 (41.3–47.8)	60.7 (58.4–62.9)	70.7 (68.8–72.5)	63.7 (62.3–65.0)
Treatment among people who were aware about their hypertension	58.4 (49.0–67.2)	68.9 (65.1–72.6)	71.9 (69.1–74.4)	70.8 (68.6–72.9)	38.4 (33.7–43.3)	64.9 (62.0–67.7)	80.1 (78.2–81.9)	71.5 (69.8–73.1)
Control among people who were treated for hypertension	34.5 (23.8–47.0)	31.2 (26.9–35.9)	34.1 (30.9–37.5)	33.2 (30.6–35.9)	43.9 (36.2–51.9)	45.0 (41.3–48.7)	49.0 (46.3–51.6)	47.6 (45.5–49.7)
**Women**	**35–44 years (*n*** **= 1901)**	**45–54 years (*n*** **= 5946)**	**55–64 years (*n*** **= 6958)**	**Total (*n*** **= 14,805)**	**40 years (*n*** **= 3657)**	**50 years (*n*** **= 4084)**	**60 years (*n*** **= 4051)**	**Total (n = 11,792)**
	**% (95% CI)**	**% (95% CI)**	**% (95% CI)**	**% (95% CI)**	**% (95% CI)**	**% (95% CI)**	**% (95% CI)**	**% (95% CI)**
Prevalence of hypertension in the population	15.6 (14.0–17.5)	33.6 (32.4–34.8)	43.7 (42.5–44.9)	35.8 (35.1–36.6)	11.0 (10.1–12.1)	29.6 (28.2–31.0)	47.9 (46.4–49.5)	28.6 (27.7–29.5)
Hypertensive people who were aware about their hypertension	24.4 (19.5–30.1)	40.3 (38.2–42.5)	49.5 (47.7–51.2)	44.3 (42.9–45.6)	56.6 (51.6–61.4)	65.4 (62.7–68.1)	74.8 (72.9–76.7)	69.1 (67.5–70.6)
Treatment among people who were aware about their hypertension	47.8 (35.6–60.1)	69.3 (66.0–72.3)	74.5 (72.2–76.6)	71.7 (69.8–73.5)	48.6 (42.0–55.3)	69.7 (66.4–72.8)	82.1 (80.0–84.0)	75.9 (74.1–77.6)
Control among people who were treated for hypertension	16.8 (7.1–34.6)	38.4 (34.4–42.4)	37.7 (34.9–40.6)	37.6 (35.3–39.9)	54.3 (44.7–63.5)	58.5 (54.4–62.6)	59.6 (56.8–62.3)	58.7 (56.5–61.0)

**Fig. 1:**
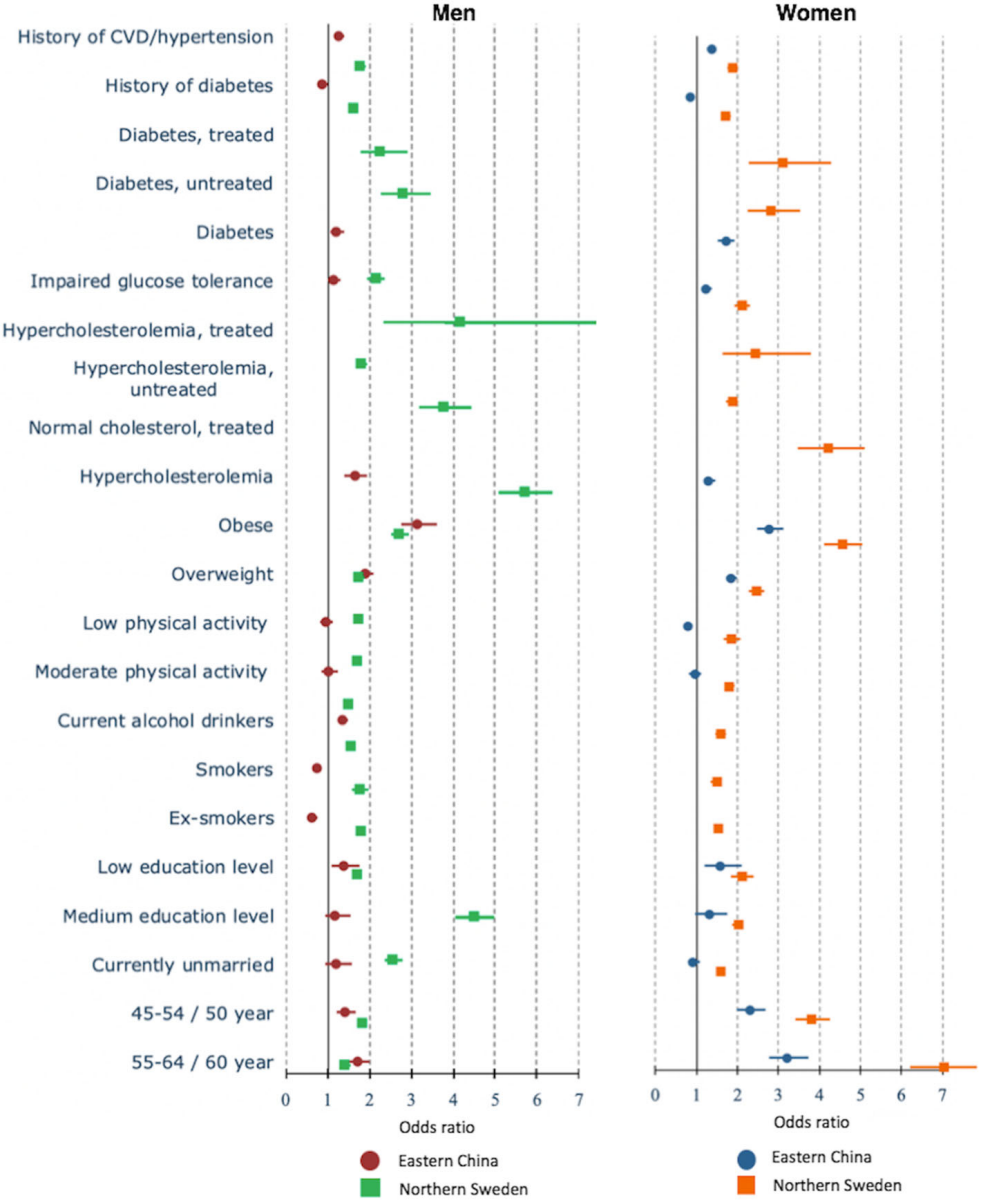
The determinants of prevalence of hypertension among men (left) and women (right) in multi-variable analyses in Eastern China and Northern Sweden

Our study revealed that men and women in both countries who were older and had low education levels, were overweight/obese, or had hypercholesterolaemia, impaired glucose tolerance or diabetes and family histories of hypertension or CVDs contributed positively to the ***prevalence of hypertension*** (Fig. 1**,** Additional file 1). Lower levels of physical activity increased the odds of having hypertension by about 20%, only among Swedish men and women (*p* < 0.05). In both countries, alcohol consumption was associated with the prevalence of hypertension among men.

**Fig. 2:**
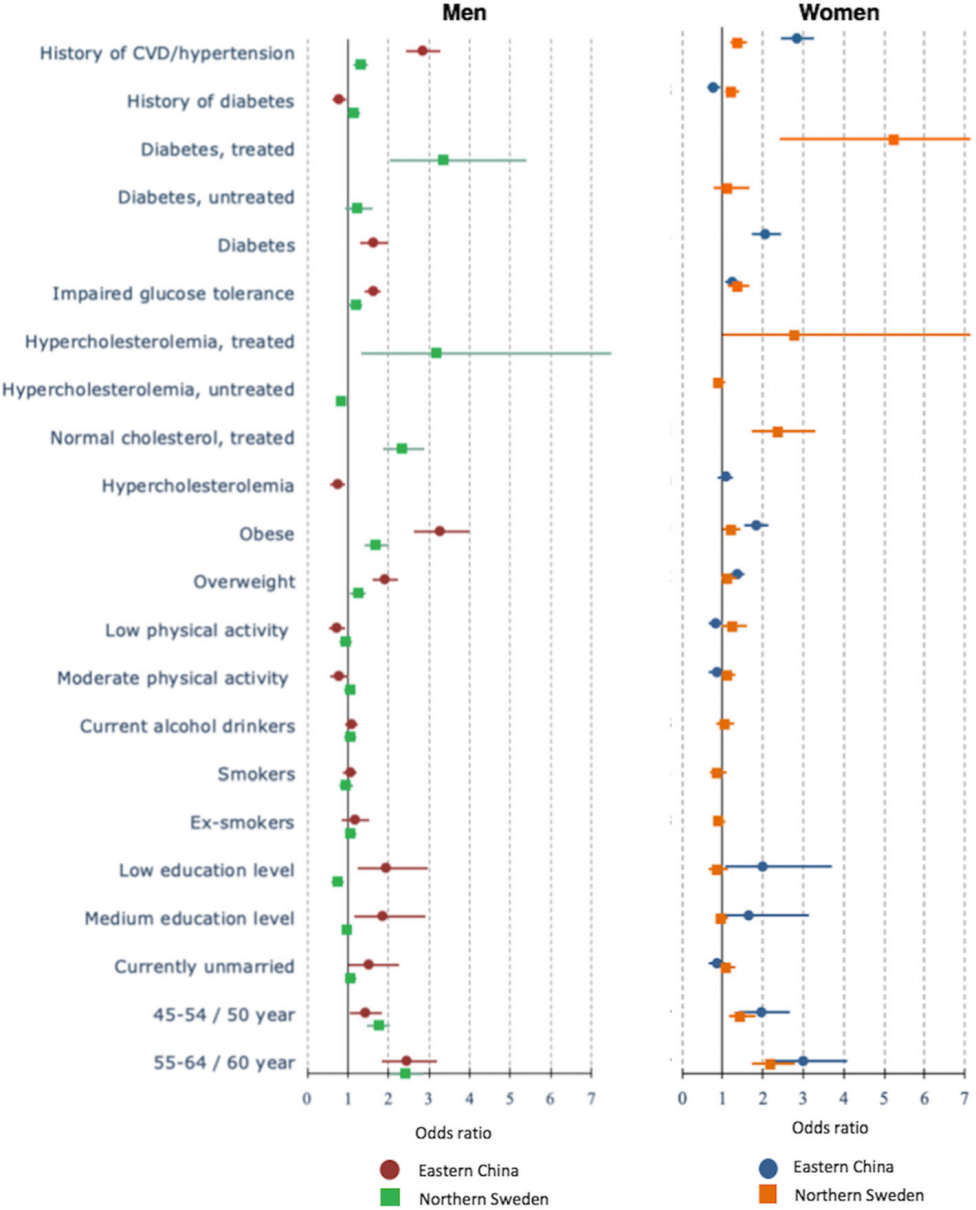
The determinants of awareness of hypertensive men (left) and women (right) in multi-variable analyses in Eastern China and Northern Sweden

The factors associated with ***awareness of hypertension*** among men and women in both countries included: older age, being overweight and obese, having an impaired glucose tolerance or diabetes and a family history of hypertension or CVDs (*p* < 0.05) (Fig. 2, Additional file 1). Having a family history of diabetes increased the odds of being diagnosed with high blood pressure by 16% among Swedish men (adjusted odds ratio/aOR = 1.16; 95%CI = 1.01–1.35), but not among Swedish women. In contrast, Chinese participants with a family history of diabetes had lower odds of being aware of their hypertension (p < 0.05). Swedish men and women who were being treated for hypercholesterolemia and diabetes had significantly higher odds of being aware of their hypertension. A low education level increased the odds of being aware of hypertension among Chinese men and women. Physical inactivity, hypercholesterolaemia and a family history of diabetes lowered the odds of being diagnosed with hypertension among Chinese men.

**Fig. 3:**
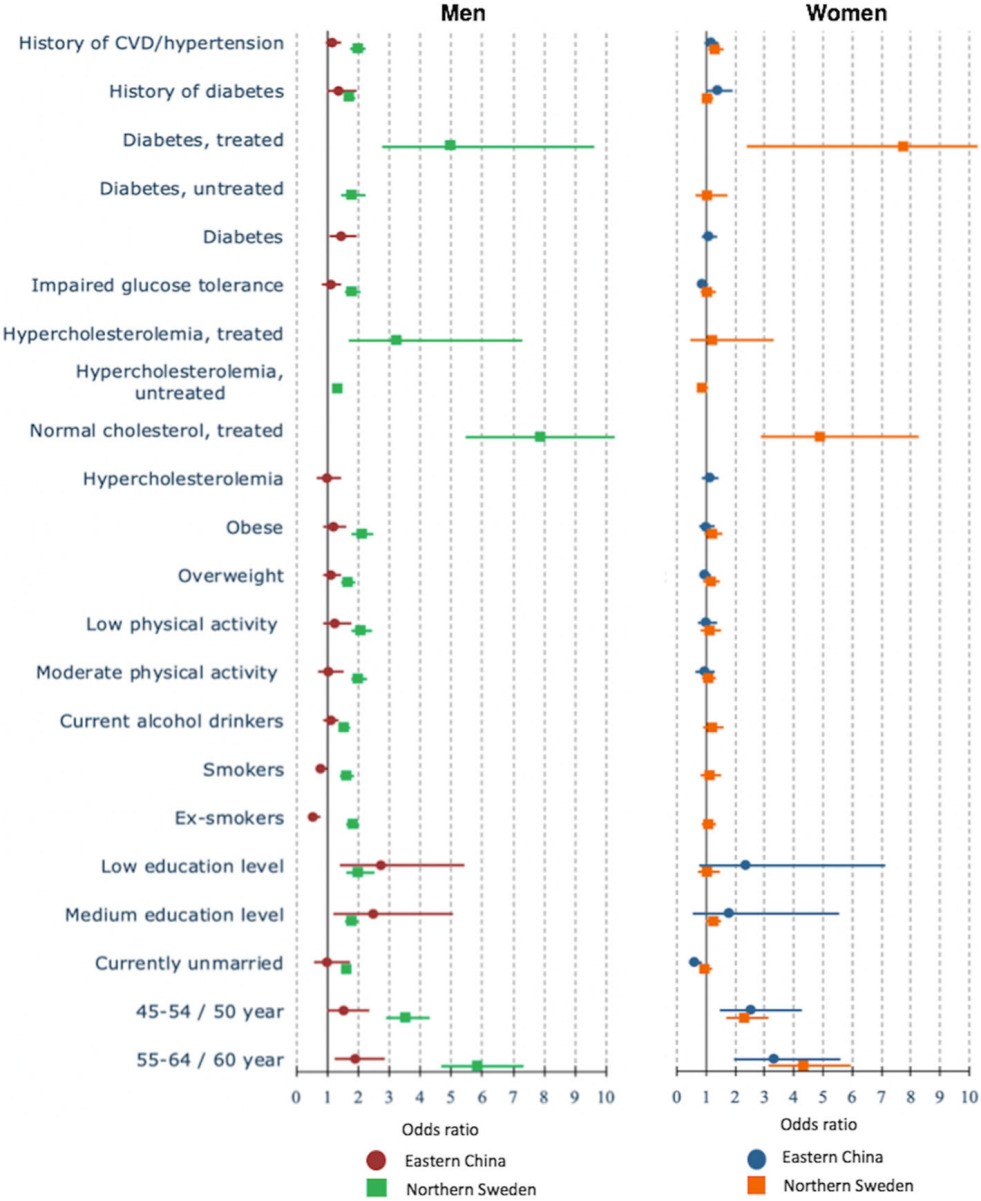
The determinants of treatment of hypertension among men (left) and women (right) in multi-variable analyses in Eastern China and Northern Sweden

The only mutual factor that increased the odds of ***treatment of hypertension*** among men and women in both countries was older age (*p* < 0.05) (Fig. 3, Additional file 1). Having a medium and low education increased the odds of being treated for hypertension only among Chinese men (aOR = 2.48, 95%CI = 1.21–5.07 and aOR = 2.74, 95%CI = 1.38–5.43, respectively). Having diabetes was associated with higher odds for hypertension treatment in China and Sweden, but the association was not significant among Chinese women. In Sweden, the odds of being treated for hypertension was higher in both men and women who were treated for high cholesterol and diabetes (*p* < 0.05). In China, hypercholesterolemia was not significantly associated with the odds of being treated for hypertension.

**Fig. 4:**
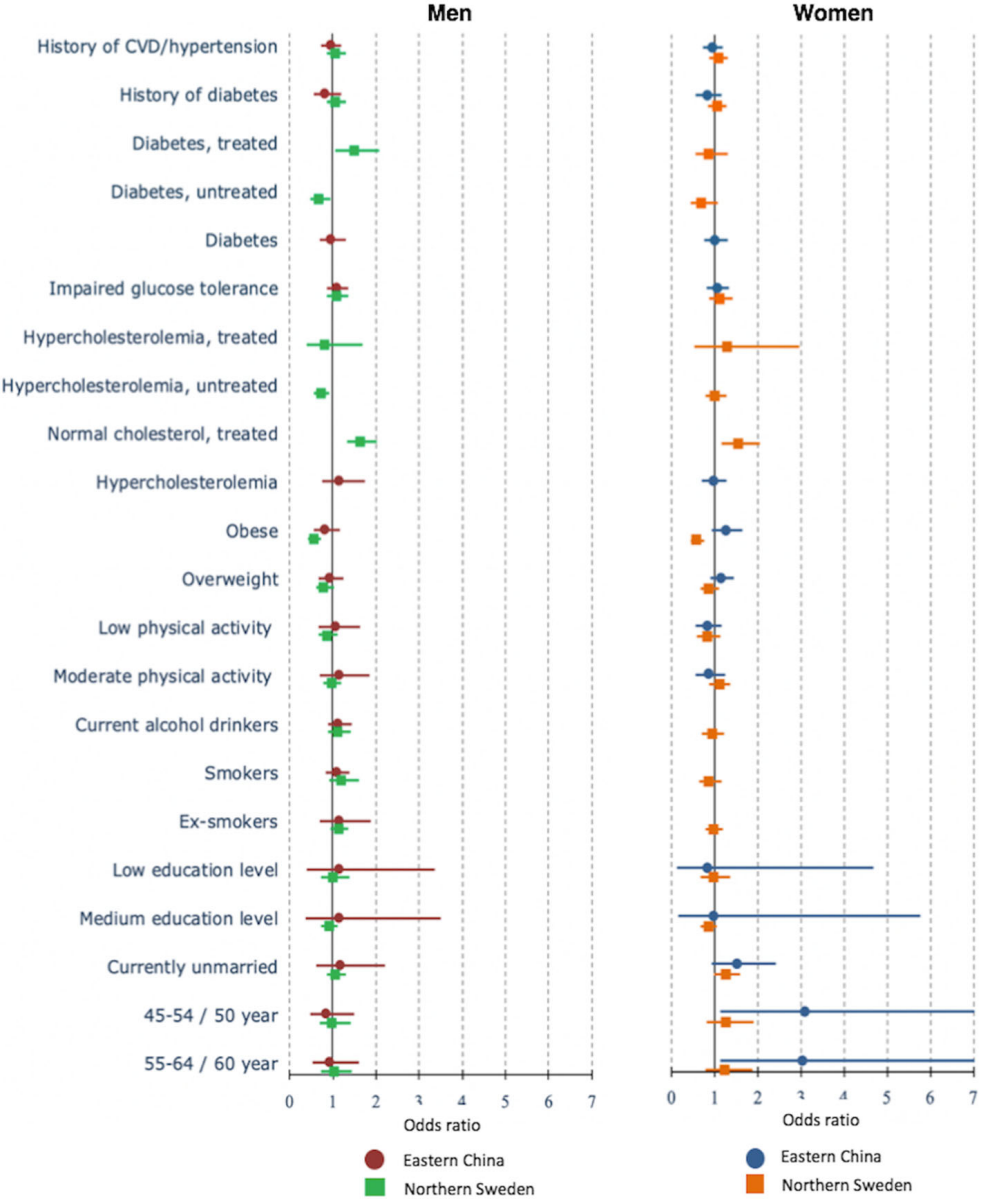
The determinants of control for hypertension among men (left) and women (right) in multi-variable analyses in Eastern China and Northern Sweden

None of the sociodemographic, biological factors and risk behaviours was significantly associated with the ***control of hypertension*** in China (Fig. 4, Additional file 1). Not being obese and having proper control of high cholesterol through medication significantly increased the odds of controlling hypertension among men and women in Sweden. Among Swedish men with diabetes treatment, the odds of controlling hypertension were higher (aOR = 1.46, 95%CI = 1.04–2.03).

## Discussions

This study reveals a few key findings. First, the prevalence of hypertension was higher among Swedish men than Chinese men, while the contrary was true for the respective women. Second, Swedish men and women were more likely to be aware, to get treatment for and to have their hypertension controlled than were their Chinese counterparts. In Sweden, men have a higher prevalence of hypertension but less awareness of and less treatment and control for it than their female counterparts. In China, men also have a higher prevalence of hypertension, but they are more aware of their hypertension, have about the same level of hypertension treatment, but have less control over it compared with Chinese women.

These observed gender differences in the prevalence, awareness, treatment and control for hypertension might reflect the different propensities men and women have to develop hypertension when its onset is related to biological mechanisms and physiological factors [30–32], as well as the differences in health literacy levels which might influence hypertension outcomes [33], health-care-seeking [34] and adherence to hypertensive treatments [35].. The gender differences should be explored in future research using qualitative designs to improve our understanding and knowledge about the underlying drivers. This knowledge will serve as a guide for developing gender-sensitive guidelines and interventions for the prevention and management of hypertension [32].

The similar low level of awareness of hypertension we observed in China has also been reported in other low- and middle-income countries [36]. As one of the major silent killers, hypertension often goes unnoticed until end-organ damage occurs, at which point mortality related to hypertension [37] cannot usually be prevented. These findings point to the importance of improving the capacity of health systems to detect and treat hypertensive individuals at an early stage. It is also essential to ensure compliance and adherence to this life-long treatment. Considering a significantly large proportion of the hypertensive patients under medication went uncontrolled, the national hypertension control programme should enhance the programme to improve adherence to treatment among patients with hypertension. Failure to promote early detection and management of hypertension will contribute to the increase of NCDs related to hypertension [37].

The presence of impaired glucose tolerance or diabetes and a family history of hypertension or CVDs were associated with higher odds for developing or being diagnosed with hypertension. Co-morbidity between hypertension and diabetes has been reported previously and significantly increases the risk of cardiovascular events and chronic renal failure [38]. Lack of physical activity and overweightness/obesity, both of which are modifiable, are significant factors relating to hypertension [39–41]. Hence, either could serve as an entry point for intervention programmes to control hypertension in the population.

A key strength of our study is that it is based on a large sample of the adult population in eastern China and northern Sweden. Both surveys were conducted using different sampling methods and instruments, hence harmonisation of the datasets was undertaken to ensure the comparability of the variables included in the study. Some limitations need to be considered in the interpretation of the results. The study is based on cross-sectional datasets, prohibiting any causal interpretation of the results. The blood pressure measurement was carried out on a single occasion; hence a clinical diagnosis of hypertension - which requires measurements from multiple occasions [42] - could not be established in this population-based study. In both countries, participants with elevated blood pressure were referred to their primary care doctor for confirmation of the hypertension diagnosis and treatment. Different methodologies used for measuring blood glucose and risk behaviours, such as smoking and physical activity, should be taken into account in considering their potential confounding effects in the analysis. As the analysis was performed separately for each country, we perceived that the lack of comparability of these confounding variables were less problematic. Last, information about treatment for diabetes and cholesterol was not available in China, which therefore precluded an in-depth understanding of the effect of treatment for diabetes and cholesterol on the burden of hypertension in China.

Future research should aim to develop interventions to promote the awareness of hypertension and adherence to hypertension treatments. Sweden has been actively reducing hypertension in its population for decades through public health policies on salt reduction, routine screening for hypertension, an integrated health intervention programme and widely-available diagnosis and treatment of hypertension [43]. In China, a national campaign for reducing salt intake started in 2006 through the National Healthy Lifestyle Campaign [44]. Multisectoral intervention to tackle sedentary lifestyles, obesity and diabetes, which are prevalent in both settings [45], should also be developed. A recent systematic review reveals several efficacious intervention programmes for improving the adherence to medication by hypertension patients in China [46]. The role of gender and the needs for developing gender-specific blood pressure guidelines should be studied further [32]. Such knowledge should be implemented and evaluated in real-world settings to ascertain the effectiveness of these interventions. Cross-national intervention design, implementation and evaluation can provide a better understanding of how an effective intervention works well in one setting but not in the other, thereby providing new knowledge that can be used to help in developing and tailoring an effective health intervention for another context. Such implementation research needs to be promoted to achieve better implementation of hypertension prevention and control programmes globally [47].

## Conclusions

This study shows the age and gender differences in the prevalence, awareness, treatment and control of hypertension among adults in China and Sweden. Multisectoral intervention should be developed to address the increasing burden of sedentary lifestyle, overweight and obesity, and diabetes; all of which are closely linked to the prevention and control of hypertension. Development and implementation of the gender- and context-specific interventions for the prevention and control of hypertension facilitates an understanding with regard to implementation barriers and facilitators.

## Supplementary Information

**Additional file 1.**

## Data Availability

The Swedish dataset generated and/or analysed are not publicly available due to the restriction of access from the Västerbotten Region, which owns the dataset. The Shanghai survey data are available upon reasonable request. The Shanghai data are not open access but can be shared under conditions of collaboration and endowment. Collaboration research projects are encouraged. Please contact the Principal Investigator of the Shanghai survey: Professor Genming Zhao (gmzhao@shmu.edu.cn).

## Abbreviations

BMI: Body Mass Index
CVDs: Cardiovascular diseases
DBP: Diastolic Blood Pressure
IPAQ: International Physical Activity Questionnaire
NCD: Non-Communicable Disease
PHC: Primary Health Care
SBP: Systolic Blood Pressure
VIP: Västerbotten Intervention Program
WGOC: Working Group on Obesity in China
WHO: World Health Organization
