# Correction to: Gender differences and determinants of prevalence, awareness, treatment and control of hypertension among adults in China and Sweden

**DOI:** 10.1186/s12889-020-10009-8

**Published:** 2021-01-04

**Authors:** Ailiana Santosa, Yue Zhang, Lars Weinehall, Genming Zhao, Na Wang, Qi Zhao, Weibing Wang, Nawi Ng

**Affiliations:** 1 Department of Epidemiology and Global Health, Faculty of Medicine, Umeå University, 90187 Umeå, Sweden; 2 Global Public Health, School of Public Health and Community Medicine, Institution of Medicine, Sahlgrenska Academy, University of Gothenburg, 41390 Gothenburg, Sweden; 3 School of Public Health, Key Laboratory of Public Health Safety of Ministry of Education, Fudan University, Shanghai, 200032 China

**Correction to: BMC Public Health 20, 1763 (2020)**

**https://doi.org/10.1186/s12889-020-09862-4**

It was highlighted that the original article [1] contained the wrong Figs. 1, 2, 3 and 4. This Correction article shows the correct Figs. 1, 2, 3 and 4. The original article has been updated.

**Fig. 1:**
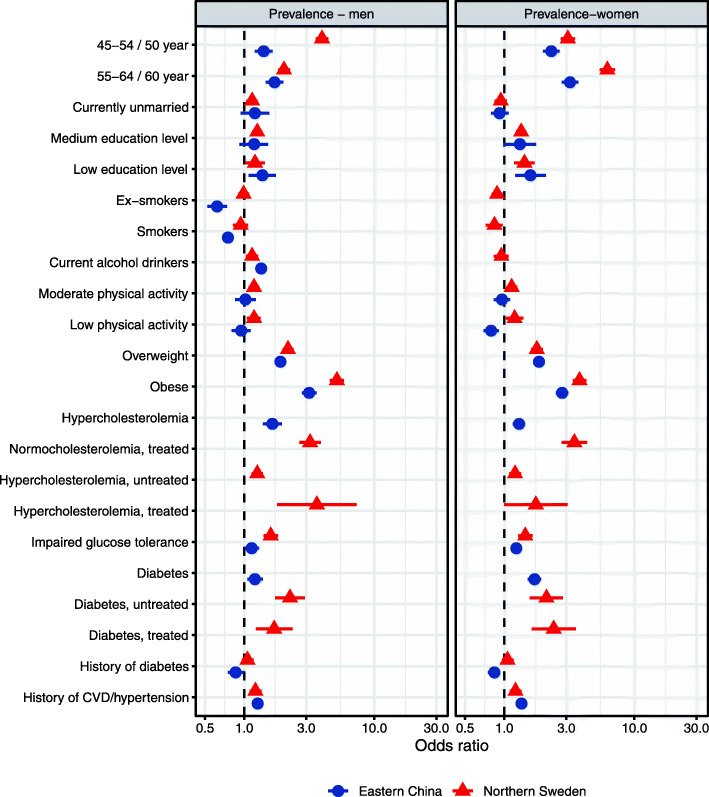
The determinants of prevalence of hypertension among men (left) and women (right) in multi-variable analyses in Eastern China and Northern Sweden

**Fig. 2:**
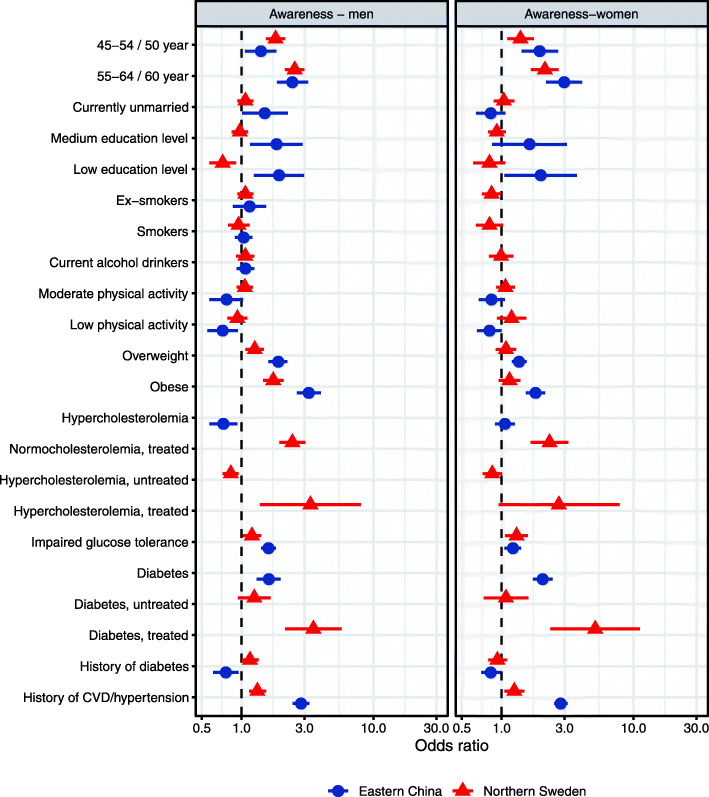
The determinants of awareness of hypertensive men (left) and women (right) in multi-variable analyses in Eastern China and Northern Sweden

**Fig. 3:**
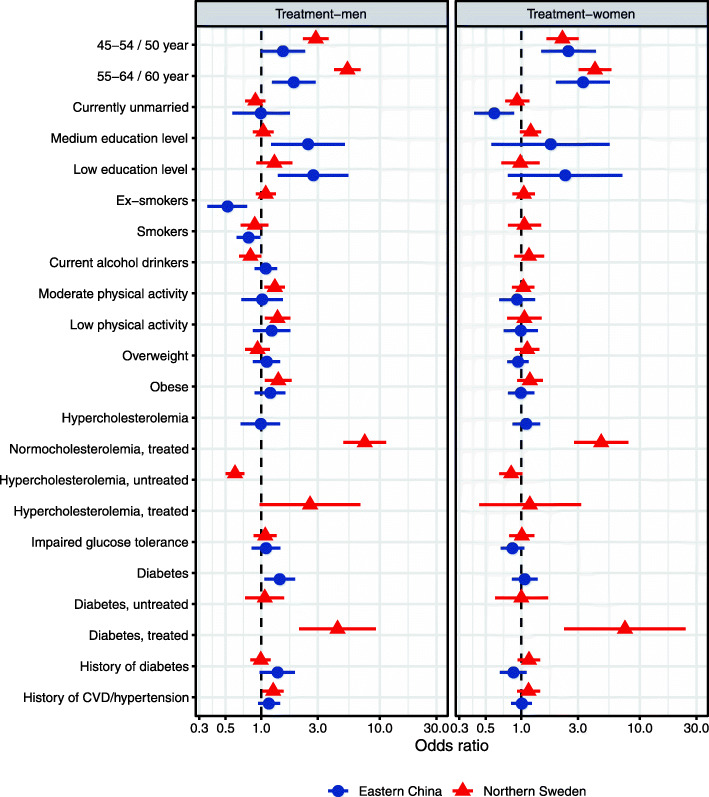
The determinants of treatment of hypertension among men (left) and women (right) in multi-variable analyses in Eastern China and Northern Sweden

**Fig. 4:**
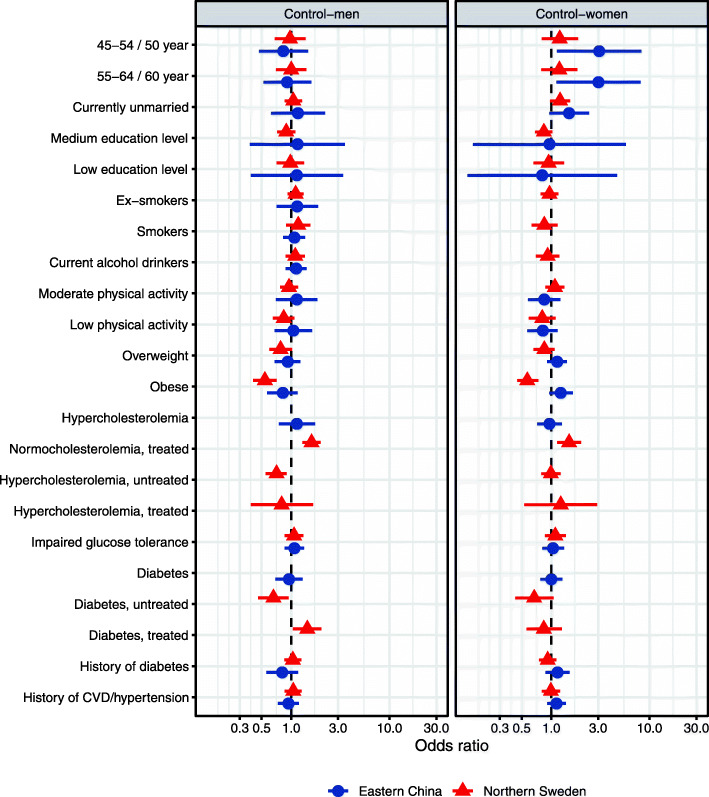
The determinants of control for hypertension among men (left) and women (right) in multi-variable analyses in Eastern China and Northern Sweden
