# Supplementary material for: Gender differences and determinants of prevalence, awareness, treatment and control of hypertension among adults in China and Sweden
Source: BMC Public Health. 2020 Nov 23;20:1763. doi: 10.1186/s12889-020-09862-4 (PMC7685617; doi:10.1186/s12889-020-09862-4)
Supplement: Supplementary file 1 — Additional file 1. [file 12889_2020_9862_MOESM1_ESM.docx]

**Appendix 1. The determinants of the prevalence and awareness of hypertension**

**among men in Eastern China and Northern Sweden**

| **Variables** | | **Prevalence** | | **Awareness** | |
| --- | --- | --- | --- | --- | --- |
|  |  | **Eastern China** | **Northern Sweden** | **Eastern China** | **Northern Sweden** |
|  |  | **aOR (95%CI)** | **aOR (95%CI)** | **aOR (95%CI)** | **aOR (95%CI)** |
| Age | |  |  |  |  |
|  | 45-54 year / 50 year | 1.41***  (1.20-1.65) | 2.02***  (1.82-2.25) | 1.40*  (1.06-1.84) | 1.81***  (1.53-2.14) |
|  | 55-64 year / 60 year | 1.71***  (1.46-2.00) | 3.96***  (3.54-4.43) | 2.43***  (1.85-3.20) | 2.53***  (2.13-3.00) |
| Marital Status | |  |  |  |  |
|  | Currently unmarried | 1.21  (0.94-1.56) | 1.15**  (1.04-1.27) | 1.50*  (1.01-2.25) | 1.07  (0.93-1.23) |
| Education level | |  |  |  |  |
|  | Medium (10-12) | 1.19  (0.92-1.53) | 1.26***  (1.15-1.38) | 1.84*  (1.16-2.91) | 0.97  (0.84-1.12) |
|  | Low (up to 9) | 1.38*  (1.08-1.75) | 1.21*  (1.02-1.44) | 1.92*  (1.24-2.98) | 0.72**  (0.57-0.91) |
| Smoking | |  |  |  |  |
|  | Ex-smokers | 0.62***  (0.52-0.74) | 0.99  (0.90-1.09) | 1.15  (0.86-1.54) | 1.07  (0.93-1.23) |
|  | Current smokers | 0.75***  (0.68-0.83) | 0.94  (0.82-1.07) | 1.04  (0.89-1.21) | 0.95  (0.79-1.15) |
| Current alcohol drinkers | |  |  |  |  |
|  | Yes | 1.35***  (1.23-1.48) | 1.15*  (1.03-1.28) | 1.07  (0.92-1.25) | 1.07  (0.91-1.25) |
| Physical activity | |  |  |  |  |
|  | Moderate level | 1.02  (0.85-1.23) | 1.19***  (1.08-1.31) | 0.77  (0.57-1.03) | 1.06  (0.92-1.22) |
|  | Low level | 0.95  (0.80-1.12) | 1.19**  (1.05-1.34) | 0.72*  (0.55-0.94) | 0.93  (0.78-1.11) |
| Body mass index | |  |  |  |  |
|  | Overweight | 1.90***  (1.73-2.09) | 2.17***  (1.97-2.40) | 1.90***  (1.60-2.23) | 1.26**  (1.07-1.48) |
|  | Obese | 3.16***  (2.77-3.61) | 5.16***  (4.56-5.83) | 3.24***  (2.63-3.99) | 1.74***  (1.46-2.09) |
| Cholesterol level | |  |  |  |  |
| Hypercholesterolemia | | 1.65***  (1.39-1.95) | NA | 0.73*  (0.57-0.93) | NA |
|  | Normal cholesterol, treated | NA | 3.21***  (2.65-3.89) | NA | 2.43***  (1.94-3.05) |
|  | Hypercholesterolemia, untreated | NA | 1.26***  (1.14-1.39) | NA | 0.83**  (0.72-0.95) |
|  | Hypercholesterolemia, treated | NA | 3.62***  (1.79-7.32) | NA | 3.33**  (1.38-8.02) |
| Fasting blood glucose | |  |  |  |  |
| Impaired glucose tolerance | | 1.14*  (1.01-1.30) | 1.60***  (1.41-1.82) | 1.60***  (1.41-1.82) | 1.20*  (1.02-1.41) |
| Diabetes | | 1.21***  (1.06-1.39) | NA | 1.61***  (1.30-1.98) | NA |
|  | Diabetes, untreated | NA | 2.25***  (1.73-2.92) | NA | 1.25  (0.94-1.66) |
|  | Diabetes, treated | NA | 1.70**  (1.23-2.36) | NA | 3.51***  2.14-5.74) |
| Family history | |  |  |  |  |
|  | Diabetes | 0.86*  (0.75-0.99) | 1.06  (0.96-1.18) | 0.76*  (0.61-0.95) | 1.16*  (1.01-1.35) |
|  | CVD / hypertension | 1.27*  (1.16-1.39) | 1.22***  (1.10-1.36) | 2.83*  (2.44-3.27) | 1.32***  (1.14-1.54) |

Notes: * p<0.05, ** p<0.01, *** p<0.05. aOR=adjusted odds ratio; CI=confidence interval. Reference groups are age 35-44 in China or 40 year in Sweden, married, high education level, never smoke, never drink alcohol or drink at most four times per month, high physical activity, underweight and normal weight, total cholesterol <6.2 mmol/L, no diabetes, and no family history of diabetes or CVD/hypertension.

**Appendix 2. The determinants of the prevalence and awareness of hypertension**

**among women in Eastern China and Northern Sweden**

| **Variables** | | **Prevalence** | | **Awareness** | |
| --- | --- | --- | --- | --- | --- |
|  |  | **Eastern China** | **Northern Sweden** | **Eastern China** | **Northern Sweden** |
|  |  | **aOR (95%CI)** | **aOR (95%CI)** | **aOR (95%CI)** | **aOR (95%CI)** |
| Age | |  |  |  |  |
|  | 45-54 year / 50 year | 2.30***  (1.98-2.67) | 3.08***  (2.71-3.51) | 1.95*  (1.42-2.68) | 1.39**  (1.10-1.76) |
|  | 55-64 year / 60 year | 3.20***  (2.76-3.72) | 6.20***  (5.41-7.10) | 2.98*  (2.18-4.08) | 2.13***  (1.67-2.71) |
| Marital Status | |  |  |  |  |
|  | Currently unmarried | 0.92  (0.79-1.08) | 0.94  (0.84-1.05) | 0.83  (0.64-1.07) | 1.04  (0.87,1.25) |
| Education level | |  |  |  |  |
|  | Medium (10-12) | 1.32  (0.98-1.76) | 1.35***  (1.23-1.48) | 1.63  (0.85-3.13) | 0.92  (0.79-1.08) |
|  | Low (up to 9) | 1.59***  (1.21-2.09) | 1.43***  (1.19-1.71) | 1.98*  (1.05-3.72) | 0.81  (0.61-1.07) |
| Smoking | |  |  |  |  |
|  | Ex-smokers | NA | 0.88*  (0.80-0.97) | NA | 0.84*  (0.71-0.99) |
|  | Current smokers | NA | 0.84*  (0.72-0.97) | NA | 0.81  (0.64-1.03) |
| Current alcohol drinkers | |  |  |  |  |
|  | Yes | NA | 0.95  (0.83-1.08) | NA | 0.99  (0.80-1.23) |
| Physical activity | |  |  |  |  |
|  | Moderate level | 0.96  (0.83-1.11) | 1.14**  (1.04-1.26) | 0.84  (0.67-1.06) | 1.07  (0.91-1.26) |
|  | Low level | 0.79*  (0.69-0.91) | 1.20*  (1.03-1.40) | 0.81  (0.65-1.00) | 1.19  (0.92-1.54) |
| Body mass index | |  |  |  |  |
|  | Overweight | 1.85***  (1.71-1.99) | 1.78***  (1.61-1.98) | 1.36*  (1.20-1.55) | 1.08  (0.90-1.29) |
|  | Obese | 2.78***  (2.49-3.11) | 3.79***  (3.37-4.27) | 1.81*  (1.53-2.14) | 1.15  (0.95-1.39) |
| Cholesterol level | |  |  |  |  |
| Hypercholesterolemia | | 1.30***  (1.16-1.45) | NA | 1.06  (0.89-1.26) | NA |
|  | Normal cholesterol, treated | NA | 3.46***  (2.76-4.34) | NA | 2.31***  (1.66-3.21) |
|  | Hypercholesterolemia, untreated | NA | 1.21***  (1.09-1.35) | NA | 0.85  (0.72-1.01) |
|  | Hypercholesterolemia, treated | NA | 1.75*  (1.00-3.07) | NA | 2.72  (0.95-7.85) |
| Fasting blood glucose | |  |  |  |  |
| Impaired glucose tolerance | | 1.24***  (1.13-1.36) | 1.45***  (1.28-1.65) | 1.22*  (1.05-1.41) | 1.30*  (1.06-1.58) |
| Diabetes | | 1.71***  (1.51-1.92) | NA | 2.05*  (1.73-2.44) | NA |
|  | Diabetes, untreated | NA | 2.11***  (1.57-2.82) | NA | 1.08  (0.73-1.60) |
|  | Diabetes, treated | NA | 2.40***  (1.62-3.55) | NA | 5.13***  (2.34-11.2) |
| Family history of diabetes | |  |  |  |  |
|  | Diabetes | 0.84*  (0.75-0.93) | 0.96  (0.86-1.06) | 0.83*  (0.70-0.98) | 0.93  (0.79-1.10) |
|  | CVD/hypertension | 1.36***  (1.26-1.46) | 1.22***  (1.09-1.35) | 2.81*  (2.50-3.17) | 1.25*  (1.05-1.49) |

Notes: * p<0.05, ** p<0.01, *** p<0.05. aOR=adjusted odds ratio; CI=confidence interval. Reference groups are age 34-44 in China or 40 year in Sweden, married, high education level, never smoke, never drink alcohol or drink at most four times per month, high physical activity, underweight or normal weight, total cholesterol <6.2 mmol/L, no diabetes, and no family history of diabetes or CVD/hypertension

**Appendix 3. The determinants of treatment and control of hypertension**

**among men in Eastern China and Northern Sweden**

| **Variables** | | **Treatment** | | **Control** | |
| --- | --- | --- | --- | --- | --- |
|  |  | **Eastern China** | **Northern Sweden** | **Eastern China** | **Northern Sweden** |
|  |  | **aOR (95%CI)** | **aOR (95%CI)** | **aOR (95%CI)** | **aOR (95%CI)** |
| Age | |  |  |  |  |
|  | 45-54 year / 50 year | 1.52  (0.98-2.34) | 2.88***  (2.24-3.70) | 0.83  (0.47-1.49) | 0.97  (0.68-1.40) |
|  | 55-64 year / 60 year | 1.88*  (1.23-2.87) | 5.33***  (4.12-6.88) | 0.91  (0.52-1.60) | 1.00  (0.70-1.43) |
| Marital Status | |  |  |  |  |
|  | Currently unmarried | 0.99  (0.57-1.74) | 0.89  (0.73-1.08) | 1.17  (0.62-2.21) | 1.05  (0.86-1.29) |
| Education level | |  |  |  |  |
|  | Medium (10-12) | 2.48*  (1.21-5.07) | 1.04  (0.85-1.27) | 1.16  (0.38-3.51) | 0.89  (0.72-1.10) |
|  | Low (up to 9) | 2.74*  (1.38-5.43) | 1.29  (0.91-1.83) | 1.14  (0.39-3.37) | 0.98  (0.71-1.35) |
| Smoking | |  |  |  |  |
|  | Ex-smokers | 0.52***  (0.35-0.76) | 1.09  (0.90-1.33) | 1.15  (0.71-1.88) | 1.11  (0.92-1.34) |
|  | Current smokers | 0.78*  (0.62-0.98) | 0.88  (0.67-1.15) | 1.08  (0.83-1.39) | 1.18  (0.89-1.57) |
| Current alcohol drinkers | |  |  |  |  |
|  | Yes | 1.09  (0.88-1.36) | 0.81*  (0.65-1.00) | 1.12  (0.88-1.44) | 1.10  (0.88-1.38) |
| Physical activity | |  |  |  |  |
|  | Moderate level | 1.02  (0.68-1.52) | 1.30*  (1.06-1.58) | 1.14  (0.70-1.85) | 0.95  (0.77-1.18) |
|  | Low level | 1.22  (0.85-1.76) | 1.37*  (1.07-1.76) | 1.05  (0.68-1.63) | 0.84  (0.65-1.08) |
| Body mass index | |  |  |  |  |
|  | Overweight | 1.11  (0.85-1.44) | 0.93  (0.73-1.18) | 0.92  (0.68-1.24) | 0.78  (0.60-1.02) |
|  | Obese | 1.19  (0.88-1.60) | 1.39*  (1.07-1.80) | 0.82  (0.57-1.16) | 0.54***  (0.41-0.71) |
| Cholesterol level | |  |  |  |  |
| Hypercholesterolemia | | 0.99  (0.67-1.44) | NA | 1.14  (0.75-1.75) | NA |
|  | Normal cholesterol, treated | NA | 7.46***  (4.93-11.29) | NA | 1.61***  (1.30-1.99) |
|  | Hypercholesterolemia, untreated | NA | 0.60***  (0.50-0.72) | NA | 0.71**  (0.55-0.90) |
|  | Hypercholesterolemia, treated | NA | 2.58  (0.97-6.86) | NA | 0.80  (0.39-1.67) |
| Fasting blood glucose | |  |  |  |  |
| Impaired glucose tolerance | | 1.10  (0.83-1.45) | 1.08  (0.86-1.35) | 1.08  (0.86-1.35) | 1.07  (0.86-1.33) |
| Diabetes | | 1.43*  (1.06-1.93) | NA | 0.95  (0.69-1.31) | NA |
|  | Diabetes, untreated | NA | 1.07  (0.73-1.56) | NA | 0.66*  (0.46-0.94) |
|  | Diabetes, treated | NA | 4.40***  (2.09-9.26) | NA | 1.46*  (1.04-2.03) |
| Family history | |  |  |  |  |
|  | Diabetes | 1.37  (0.97-1.92) | 0.99  (0.81-1.20) | 0.81  (0.56-1.18) | 1.04  (0.85-1.27) |
|  | CVD / hypertension | 1.16  (0.94-1.44) | 1.26*  (1.02-1.54) | 0.94  (0.73-1.20) | 1.05  (0.86-1.28) |

Notes: * p<0.05, ** p<0.01, *** p<0.05. aOR=adjusted odds ratio; CI=confidence interval. Reference groups are age 35-44 in China or 40 year in Sweden, married, high education level, never smoke, never drink alcohol or drink at most four times per month, high physical activity, underweight and normal weight, total cholesterol <6.2 mmol/L, no diabetes, and no family history of diabetes or CVD/hypertension.

**Appendix 4. The determinants of treatment and control of hypertension**

**among women in Eastern China and Northern Sweden**

| **Variables** | | **Treatment** | | **Control** | |
| --- | --- | --- | --- | --- | --- |
|  |  | **Eastern China** | **Northern Sweden** | **Eastern China** | **Northern Sweden** |
|  |  | **aOR (95%CI)** | **aOR (95%CI)** | **aOR (95%CI)** | **aOR (95%CI)** |
| Age | |  |  |  |  |
|  | 45-54 year / 50 year | 2.50***  (1.47-4.26) | 2.22***  (1.63-3.04) | 3.07*  (1.14-8.27) | 1.22  (0.80-1.88) |
|  | 55-64 year / 60 year | 3.31***  (1.96-5.59) | 4.18***  (3.05-5.75) | 3.03*  (1.13-8.12) | 1.21  (0.79-1.84) |
| Marital Status | |  |  |  |  |
|  | Currently unmarried | 0.59*  (0.40-0.87) | 0.92  (0.73,1.17) | 1.52  (0.95-2.43) | 1.23  (0.97,1.56) |
| Education level | |  |  |  |  |
|  | Medium (10-12) | 1.77  (0.56-5.56) | 1.20  (0.97-1.47) | 0.96  (0.16-5.76) | 0.84  (0.68-1.03) |
|  | Low (up to 9) | 2.34  (0.77-7.13) | 0.98  (0.68-1.43) | 0.81  (0.14-4.69) | 0.94  (0.66-1.35) |
| Smoking | |  |  |  |  |
|  | Ex-smokers | NA | 1.05  (0.84-1.30) | NA | 0.96  (0.78-1.18) |
|  | Current smokers | NA | 1.06  (0.77-1.47) | NA | 0.85  (0.63-1.16) |
| Current alcohol drinkers | |  |  |  |  |
|  | Yes | NA | 1.16  (0.87-1.56) | NA | 0.92  (0.70-1.21) |
| Physical activity | |  |  |  |  |
|  | Moderate level | 0.92  (0.65-1.31) | 1.04  (0.83-1.29) | 0.85  (0.58-1.24) | 1.09  (0.87-1.36) |
|  | Low level | 0.99  (0.71-1.38) | 1.06  (0.76-1.48) | 0.82  (0.57-1.16) | 0.81  (0.59-1.11) |
| Body mass index | |  |  |  |  |
|  | Overweight | 0.94  (0.76-1.15) | 1.12  (0.88-1.42) | 1.15  (0.91-1.44) | 0.85  (0.66-1.08) |
|  | Obese | 0.99  (0.77-1.29) | 1.18  (0.92-1.52) | 1.25  (0.95-1.66) | 0.57***  (0.45-0.74) |
| Cholesterol level | |  |  |  |  |
| Hypercholesterolemia | | 1.10  (0.84-1.44) | NA | 0.96  (0.72-1.28) | NA |
|  | Normal cholesterol, treated | NA | 4.72***  (2.79-8.00) | NA | 1.52**  (1.15-2.02) |
|  | Hypercholesterolemia, untreated | NA | 0.82  (0.65-1.02) | NA | 0.99  (0.79-1.25) |
|  | Hypercholesterolemia, treated | NA | 1.18  (0.44-3.19) | NA | 1.25  (0.53-2.93) |
| Fasting blood glucose | |  |  |  |  |
| Impaired glucose tolerance | | 0.84  (0.67-1.06) | 1.01  (0.79-1.29) | 1.04  (0.81-1.35) | 1.10  (0.86-1.41) |
| Diabetes | | 1.07  (0.83-1.37) | NA | 1.00  (0.77-1.30) | NA |
|  | Diabetes, untreated | NA | 1.00  (0.60-1.68) | NA | 0.67  (0.43-1.06) |
|  | Diabetes, treated | NA | 7.49***  (2.30-24.3) | NA | 0.84  (0.56-1.28) |
| Family history of diabetes | |  |  |  |  |
|  | Diabetes | 0.86  (0.66-1.11) | 1.16  (0.93-1.44) | 1.16  (0.87-1.54) | 0.92  (0.75-1.13) |
|  | CVD/hypertension | 1.01  (0.82-1.23) | 1.15  (0.92-1.44) | 1.13  (0.91-1.41) | 0.99  (0.80-1.23) |

Notes: * p<0.05, ** p<0.01, *** p<0.05. aOR=adjusted odds ratio; CI=confidence interval. Reference groups are age 34-44 in China or 40 year in Sweden, married, high education level, never smoke, never drink alcohol or drink at most four times per month, high physical activity, underweight or normal weight, total cholesterol <6.2 mmol/L, no diabetes, and no family history of diabetes or CVD/hypertension.
